# Three Effective, Efficient, and Easily Implementable Ways to Integrate A.I. Into Medical Education

**DOI:** 10.7759/cureus.47204

**Published:** 2023-10-17

**Authors:** David Lenihan

**Affiliations:** 1 Neurology, Ponce Health Sciences University, Ponce, PRI; 2 Neurology, Ponce Health Sciences University, St. Louis, USA

**Keywords:** a.i, chatgpt, medical school education, medical education and training, artificial intelligence and education

## Abstract

As a medical school CEO who is following the development of A.I. very closely, I believe that med students are eager to adopt the possibilities that A.I. tools can deliver in their training. Not only do these students already use variations of A.I. in other areas of their lives, but they also embrace advanced technology and understand how to use it.

With the tech readiness of today’s students in mind, I have devised three recommendations for how to best infuse A.I. into medical education. This strategic guidance can deliver significant benefits to today’s tech-fluent medical school students and enhance their training in their journeys to become doctors.

## Editorial

As a medical school CEO who’s following the development of A.I. very closely, I’m convinced that today’s 20-something med students are eager to adopt the possibilities that such exciting tools as ChatGPT, Google's Bard, Baidu's Ernie, and OpenAI can deliver in their training. Not only do these students already use variations of A.I. in other areas of their lives, but they also embrace advanced technology and 100% “get” how to use it.

According to a great article by Danielle Abril in the Washington Post, “since they were tykes, [Gen Z has] been exposed to digital devices and services - the oldest of the bunch were about a year old when Google launched. As a result, they tend to be open to exploring new technologies” and “are more likely to use A.I. compared with older counterparts [1].

“They’re the first digital-native generation,” said Shaun Pichler, College of Business and Economics at California State University. “They’re used to using tech [such as A.I.] day in and day out” [1].

With the tech readiness of today’s students in mind, I have devised three recommendations for how to best infuse A.I. into medical education. This strategic guidance can deliver significant benefits to today’s tech-fluent medical school students and enhance their knowledge/skills/capabilities as our future doctors.

Teach A.I. literacy as it applies to medical education

In order for A.I. to be useful for med students, it is essential to educate them about how to frame the questions that are entered into their A.I. tools in order to get the most relevant answers to properly inform and guide their medical decisions. 

For instance, A.I. - at this point, at least - is not helpful if you are trying to teach students how to execute a particular medical procedure correctly, but it can allow students to understand how that procedure affects the physiology of the patient.

An example of this would be the mechanical process of inserting an IV. A.I. (again, as of this writing) would probably return a muddled response to the question “how do you insert an IV?” However, if the student’s question is sharpened to be “what is the flow rate of an IV on a 90 pound patient with peripheral vascular disease?”, the A.I. tool would absolutely deliver actionable data and insights.

Being fluent and comfortable in the particular language of A.I. inputs, perhaps taught in conjunction with, and consistently reviewed by, a team composed of a coder as well as medical professionals from diverse disciplines (nurses, physical therapists, nutritionists, etc.) would prepare medical students to acquire the greatest learning benefits from their A.I. tools.

Instruct students how to discern whether the info that is being served back by the A.I. tool is viable, truthful, and accurate

George Fuechsel’s timeless computer programming adage “garbage in, garbage out” (aka GIGO) also applies to A.I. tools [2].

On the entry end, the teaching of A.I. literacy would offer med school students a framework to maximize the precision of their queries in order to generate the most suitable responses from the A.I. tool. Such awareness would de-garbage-ify their inputs.

But as far as the tool’s output is concerned, students would also need to be taught to discern whether the information delivered is appropriate and trustworthy. They’d need to understand that even with a meticulously crafted query, there is still potential for the A.I. tool to serve up a response that has hints of a garbage-y-ness.

Recognizing the possibility of GISGO (a new acronym that stands for “genius in, still garbage out”) with A.I. tools will prevent faulty outputs from creating really faulty and potentially harmful medical decisions. This awareness would also solidify med students’ recognition that while A.I. can present valuable answers, it should never be regarded as an infallible and indisputable conduit to “the” answer.

Avoid trying to turn med students into coders

An article by Katie Palmer in STAT that features an interview with Erkin Ötleş, Medical School and College of Engineering at the University of Michigan, floats the idea of teaching A.I. coding to med students [3].

“There’s been a discussion of: All these [A.I.] techniques use a lot of Python programming, so we should teach medical students Python programming,” says Ötleş. “And, you know, I love Python programming, but I don’t think all my colleagues coming out of medical school should know how to program in Python [3].”

Ötleş’ observation about the non-need for medical students to learn Python is valid, but it does not take into account the blowback that would occur if A.I. coding became a mandatory part of the curriculum. If such an addition were made at Ponce Health Sciences University, for example, there would be a loud revolt by our students. The backlash would be intense, and such instruction, while possibly beneficial, would be a DOA non-starter. I imagine that a similar response would play out at other med schools.

There would be two reasons for this resistance: first, if coding instruction is added to the coursework, then a more core instruction module would need to be taken out since there are a limited number of hours for medical education teaching to happen. Second, med students do not need to know, understand, or master the mechanics of A.I. in order for them to take advantage of the usefulness that the tools can deliver. Gaining A.I. literacy (see #1 above) would provide med students with a significantly more focused and effectual way to leverage A.I. than learning Python or other A.I. languages.

I agree with Dr. Jim Woolliscroft, Learning Health Sciences Department at the University of Michigan, who said in STAT “I just think it’s important enough that [A.I.] be put on a fast track [in medical schools] rather than allowed to just develop organically [3].” Unfortunately, the slow-moving ship of medical education is simply unable to pivot as quickly as Woolliscroft believes it should in order for tomorrow’s future doctors to speedily incorporate A.I. tools into their learning.

However, medical education’s reticence to nimble upgrades does not mean that A.I. cannot gradually and carefully be woven into the curriculum. Our students are ready for it, the marketplace is demanding it, and there are ways to inject it so that it can be seamlessly merged with current coursework. The sensible steps detailed above offer an achievable gateway to its more widespread acceptance and adaptation.

## References

[1] (2023). New Gen Z graduates are fluent in AI and ready to join the workforce. https://www.washingtonpost.com/technology/2023/06/28/ai-gen-z-work/.

[2] (2023). How medical schools are missing the mark on artificial intelligence. https://www.statnews.com/2023/01/12/medical-school-artificial-intelligence-health-curriculum/.

[3] (2023). Garbage in, garbage out (GIGO). https://www.techtarget.com/searchsoftwarequality/definition/garbage-in-garbage-out#:~:text=The%20first%20recorded%20use%20of,term%20in%20the%20early%201960s.

